# Dependence of p53-deficient cells on the DHX9 DExH-box helicase

**DOI:** 10.18632/oncotarget.15889

**Published:** 2017-03-03

**Authors:** Teresa Lee, Jerry Pelletier

**Affiliations:** ^1^ Department of Biochemistry, McGill University, Montreal, Quebec, H3G 1Y6, Canada; ^2^ Department of Oncology, and McGill University, Montreal, Quebec, H3G 1Y6, Canada; ^3^ Rosalind and Morris Goodman Cancer Research Center, McGill University, Montreal, Quebec, H3G 1Y6, Canada

**Keywords:** DHX9, helicase, p53, drug target, apoptosis

## Abstract

DHX9 is a DExH-box helicase family member with key regulatory roles in a broad range of cellular processes. It participates at multiple levels of gene regulation, including DNA replication, transcription, translation, RNA transport, and microRNA processing. It has been implicated in tumorigenesis and recent evidence suggests that it may be a promising chemotherapeutic target. Previous studies have determined that DHX9 suppression elicits an apoptotic or senescence response by activating p53 signaling. Here, we show that DHX9 inhibition can also have deleterious effects in cells lacking functional p53. Loss of DHX9 led to increased cell death in p53-deficient mouse lymphomas and HCT116 human colon cancer cells, and G0/G1 cell cycle arrest in p53-deficient mouse embryonic fibroblasts. Analysis of mRNA levels for p53 transcriptional targets showed that a subset of p53 targets in the p53-null lymphomas and HCT116 cells were activated despite the absence of functional p53. This implies an alternative pathway of DHX9-mediated activation of cell death and cell cycle arrest in p53-deficient cells and supports the feasibility of targeting DHX9 in p53-deficient tumors.

## INTRODUCTION

DHX9 (also known as Nuclear DNA Helicase II (NDH II) and RNA Helicase A (RHA)) is an NTP-dependent helicase belonging to the DExH-box family of helicase proteins. DHX9 is a multi-domain protein, consisting of a core helicase domain harboring the conserved DEIH sequence, two RNA-binding domains at the N-terminus, a nuclear transport domain and a DNA-binding RGG-box at the C-terminus [1]. It is capable of unwinding a variety of substrates, including DNA, RNA, and complex polynucleotide structures [2, 3], and has been implicated in many diverse biological processes. Its functions include regulation of transcription [4–6], translation [7, 8], RNA transport [9], microRNA processing [10], and DNA replication and genome maintenance [11–14]. Over 30 interacting partners for DHX9 have been identified, in the context of its various cellular roles [15]. Due to the important regulatory role played by DHX9, there is growing evidence of its implications in human diseases such as various cancers and viral infections [15].

We previously identified DHX9 as a modifier of sensitivity to ABT-737 (an inhibitor of BCL-2 family pro-survival factors) in a mouse lymphoma model. Using *Arf*^−/−^Eμ-*Myc*/Bcl-2 mouse lymphoma cells, which overexpressed c-MYC and exogenous BCL-2 and were resistant to ABT-737, we found that suppression of DHX9 synergized with ABT-737 to reverse resistance. This was accomplished through aggravation of replicative stress and activation of p53 signaling, leading to apoptosis [16]. We subsequently investigated the effect of DHX9 suppression in non-transformed primary human diploid fibroblasts, where we demonstrated that loss of DHX9 resulted in a pronounced growth arrest and premature senescence. This resulted from inhibition of DNA replication which activated a p53-dependent stress response [17]. In both the *Arf*^−/−^Eμ-*Myc*/Bcl-2 mouse lymphoma and primary human fibroblast models, functional p53 signaling was essential for the ABT-737 synergy or senescence response.

Further exploration of the chemotherapeutic potential of targeting DHX9 has been carried out in other mouse and human cancer models. Whereas DHX9 was targeted in combination with ABT-737 treatment in the aforementioned *Arf*^−/−^Eμ-*Myc*/Bcl-2 lymphomas, it was found that loss of DHX9 on its own had a lethal effect on tumor cells where BCL-2 was not supra-elevated. In MYC-driven *TSC2^+/−^*Eμ*-Myc* lymphomas, DHX9 suppression had a straight lethal effect both *ex vivo* and *in vivo* [18]. Knockdown of DHX9 in a representative panel of human cancer cell lines, including multiple myeloma, osteosarcoma, and breast, lung, and cervical cancer cells, demonstrated that DHX9 suppression was detrimental in the majority of cancer cells [18]. In assessing the levels of various apoptotic and cell cycle proteins in the different cell lines, we noted that two of them, MDA-MB-231 breast cancer cells and HeLa cervical cancer cells, harbored a mutation in p53 or were p53-deficient. Despite the absence of functional p53, however, loss of DHX9 had a deleterious effect on both cell lines [18]. This suggested that p53 was not the only factor mediating the apoptotic effect of DHX9 suppression, and that there may be a p53-independent mechanism triggering cell death upon DHX9 suppression.

In this study, we investigate the phenomenon and underlying mechanisms of DHX9-mediated cell death and growth arrest in p53-deficient systems. We compare the consequences of DHX9 suppression in p53-wildtype and p53-deficient settings in three different *ex vivo* models: mouse lymphomas, mouse embryonic fibroblasts (MEFs), and human colon cancer cells. We demonstrate that in all three cases, loss of DHX9 leads to a reduction in cellular fitness in both p53-wildtype and p53-deficient cells. Analysis of the levels of p53 transcriptional targets in each system shows that in the absence of p53, some targets were nevertheless activated upon DHX9 suppression. Our results support the existence of a p53-independent aspect to DHX9-mediated cell death and cell cycle arrest, and highlight the value of targeting DHX9 in p53-defective tumors.

## RESULTS

### DHX9 suppression reduces cellular fitness in both p53-wildtype and p53-null settings

**Figure 1:**
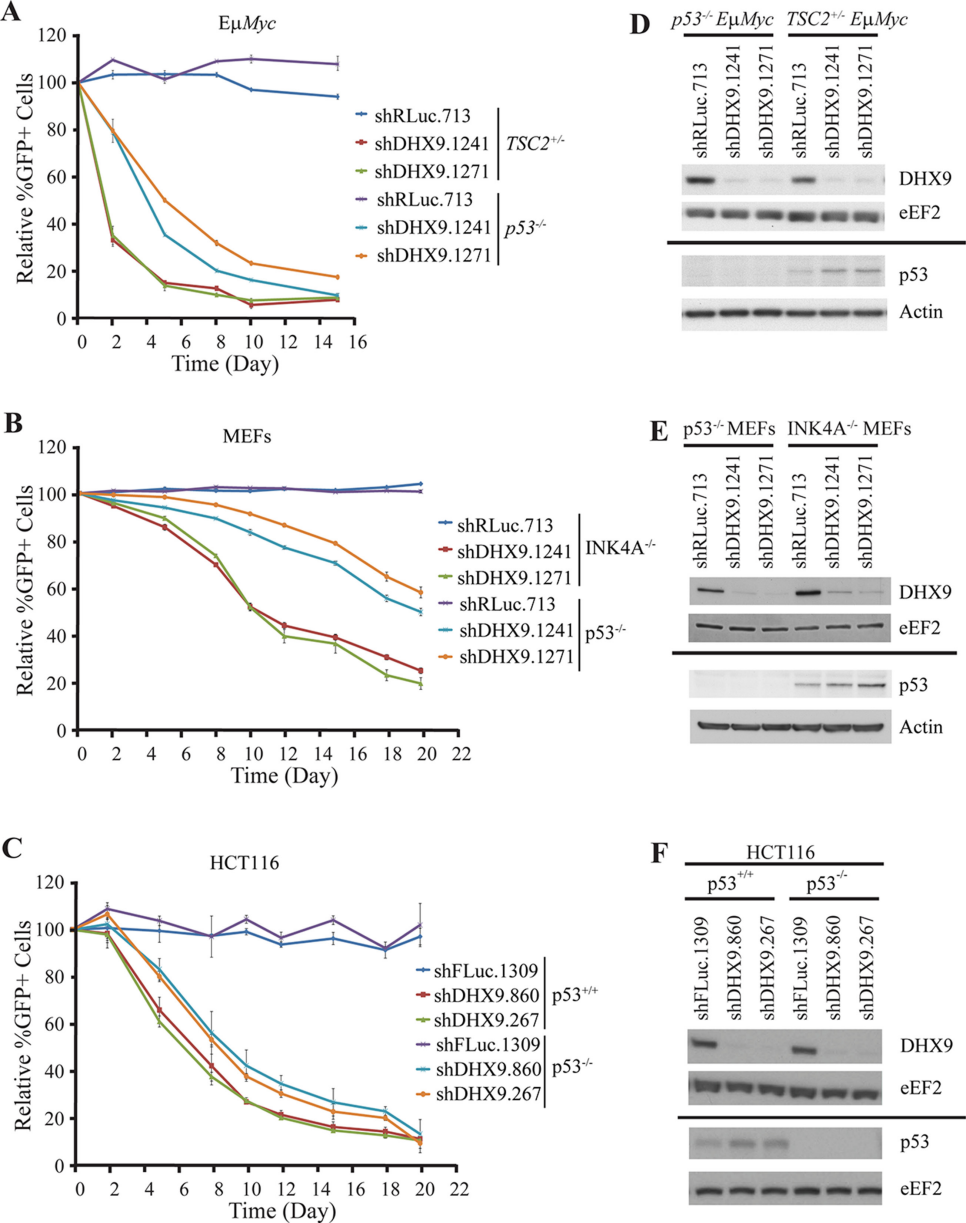
DHX9 suppression reduces cellular fitness in both p53-wildtype and p53-null systems *Ex vivo* competition assay with (**A**) *TSC2^+/−^*Eμ-*Myc* (p53^+/+^) and *p53^−/−^* Eμ-*Myc* lymphomas, (**B**) INK4A^−/−^ (p53^+/+^) and p53*^−/−^* MEFs, and (**C**) HCT116 p53^+/+^ and HCT116 p53^−/−^ cells. Cells were infected with shRNAs targeting DHX9 or a neutral control (shRLuc.713) and the relative %GFP monitored over time. The experiment was started 48 hours after the final infection (*t* = Day 0). *N* = 3 ± SEM. (**D**–**F**) Western blot analysis of extracts from the indicated cell lines. Membranes are probed with antibodies indicated to the left. Solid bar indicates that a different set of Western blots were probed.

Previous studies in both non-transformed cells and tumor models initially suggested that functional p53 signaling is essential for the cell death or senescence response resulting from DHX9 inhibition [16, 17]. Further investigation, however, demonstrated that MDA-MB-231 cells, which harbor a point mutation in p53, and HeLa cells, which are p53-deficient due to overexpression of the E6 protein from human papillomavirus type 16, also showed increased cell death upon DHX9 suppression [18]. To characterize this response, we knocked down DHX9 in p53-wildtype and p53-null settings in three different cell types. *p53^−/−^*Eμ-*Myc* lymphomas were compared to *TSC2^+/−^*Eμ-*Myc* lymphomas – the latter of which were previously characterized and shown to contain functional p53 signaling as well as being highly responsive to DHX9 suppression [18–20]. A competition assay was carried out in which cells infected with shRNAs targeting DHX9 or a neutral renilla luciferase control (shRLuc.713) were co-cultured with non-infected cells (Figure 1A). Cells harboring DHX9 shRNAs were depleted (represented by a decrease in %GFP+ cells) in both *TSC2^+/−^*Eμ-*Myc* and *p53^−/−^*Eμ-*Myc* lymphomas; however, the kinetics of the depletion was slower in the case of the *p53^−/−^*Eμ-*Myc* lymphomas (Figure 1A). This result was recapitulated in INK4A^−/−^ (p53^+/+^) and p53^−/−^ MEFs (Figure 1B). Here, shDHX9-expressing cells were depleted in both p53^+/+^ and p53^−/−^ MEFs, but the kinetics were slower in the latter compared to the former. We also examined the outcome of knocking down DHX9 in HCT116 p53^+/+^ and HCT116 p53^−/−^ cells. HCT116 p53^−/−^ cells were derived from parental HCT116 p53^+/+^ cells through disruption of both alleles of the p53 gene by homologous recombination and hence these are isogenic cell lines [21]. As with the lymphomas and MEFs, both the HCT116 p53^+/+^ and HCT116 p53^−/−^ cells exhibited depletion of GFP+ cells following DHX9 suppression (Figure 1C). Here, the kinetics of depletion are relatively similar, with the depletion in the HCT116 p53^−/−^ cells being only slightly slower than that of the HCT116 p53^+/+^ cells. The variation in kinetics is unlikely due to differences in DHX9 knockdown, which were quite similar in all three pairs of cell lines examined (Figure 1D–1F). The p53 status in all three cell types was verified by Western blot analysis, and in the p53^+/+^ scenarios, DHX9 suppression led to elevation of p53 levels (Figure 1D–1F), in agreement with prior studies [16–18]. Our results demonstrating that shDHX9-expressing cells were depleted in three independent p53-null cell lines support previous observations that DHX9 suppression can be detrimental to cells without functional p53.

Whereas p53^+/+^ and p53^−/−^ isogenic lines were used for the competition assays in HCT116 cells, the p53-wildtype and p53-null lymphomas and MEFs were not isogenic. *TSC2^+/−^*Eμ-*Myc* lymphomas and INK4A^−/−^ MEFs were used because they contain functional p53 signaling [18–20, 22]. To verify that the TSC2 status of the lymphomas does not affect the response of the cells to DHX9 suppression, we generated isogenic cell lines where TSC2 was suppressed via shRNA in either *p53^−/−^*Eμ-*Myc* lymphomas or *Arf^−/−^*Eμ-*Myc* lymphomas (which harbor functional p53). A comparison of competition assays performed in control shFLUC.1309 and shTSC2-transduced cells showed no significant difference in the depletion kinetics of GFP+ cells following DHX9 knockdown, in either the *p53^−/−^*Eμ-*Myc* or *Arf^−/−^*Eμ-*Myc* lymphomas (Supplementary Figure 1A and 1C). This demonstrates that loss of TSC2 does not affect the cellular response to DHX9 suppression.

Similarly, to eliminate the possibility that the INK4A status of the MEFs may represent a confounding factor in the DHX9 response, we generated isogenic lines by knocking out p53 via CRISPR-mediated gene-editing in INK4A^−/−^ parental cells. p53-mutated cells were selected using Nutlin-3a, and editing at the p53 locus was confirmed by T7 endonuclease cleavage, via Western blotting, and by colony formation assays (Supplementary Figure 2A–2C). A competition assay performed with control sgROSA or sgp53-transduced INK4A^−/−^ MEFs showed depletion of DHX9-expressing cells in both the control and p53-edited settings; however, the kinetics were slower in the latter compared to the former (Supplementary Figure 2D), despite the two cell lines exhibiting similar robust DHX9 knockdown levels (Supplementary Figure 2E). This difference in kinetics was comparable to the differences observed between the INK4A^−/−^ parental MEFs and the p53^−/−^ MEFs. Hence, the TSC2 or INK4A status of the cells used in this study does not appear to significantly affect the response to DHX9 suppression. The *TSC2^+/−^*Eμ-*Myc* lymphomas and INK4A^−/−^ MEFs were therefore used as control lines for the remainder of this study.

### Context-dependent effects of DHX9 suppression in p53-deficient cells

**Figure 2:**
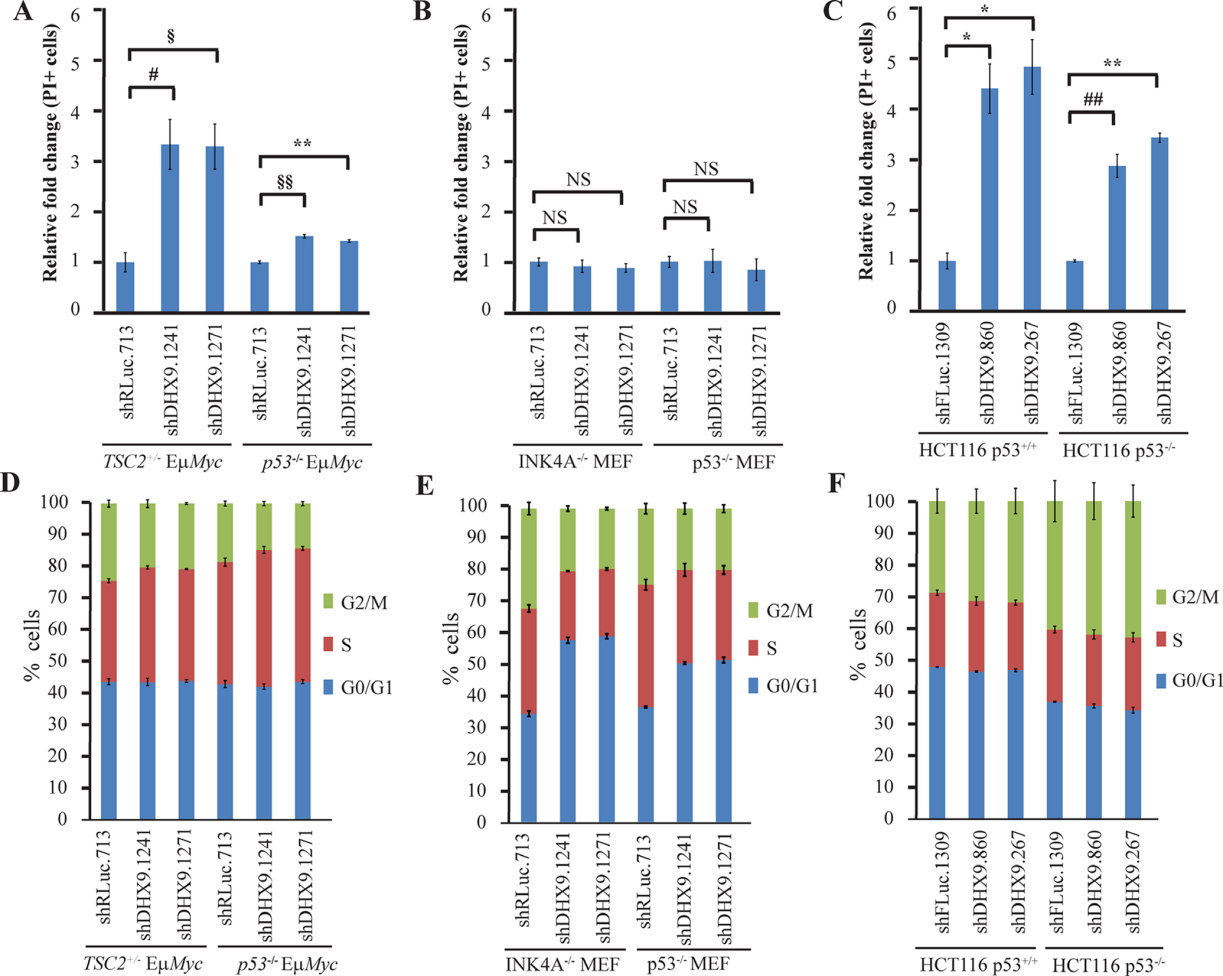
Context-dependent consequences of DHX9 suppression PI staining of (**A**) *TSC2^+/−^*Eμ-*Myc* (p53^+/+^) and *p53^−/−^* Eμ-*Myc* lymphomas, (**B**) INK4A^−/−^ (p53^+/+^) and p53^−/−^ MEFs, and (**C**) HCT116 p53^+/+^ and HCT116 p53^−/−^ cells expressing the indicated shRNAs 7 days post-infection. *N* = 3 ± SEM. ^#^*p* ≤ 0.05, ^§^*p* ≤ 0.01, *p ≤ 0.005, ^##^*p* ≤ 0.001, ^§§^*p* ≤ 0.0005, ^*^p ≤ 0.0001, NS – not significant. Cell cycle analysis of (**D**) *TSC2^+/−^*Eμ-*Myc* (p53^+/+^) and *p53^−/−^* Eμ-*Myc* lymphomas, (**E**) INK4A^−/−^ (p53^+/+^) and p53^−/−^ MEFs, and (**F**) HCT116 p53^+/+^ and HCT116 p53^−/−^ cells expressing the indicated shRNAs 10 days post-infection. *N* = 3 ± SEM.

To gain insight into the underlying causes for the difference in kinetics observed between p53-wildtype and p53-deficient systems, we quantitated the levels of cell death that ensued following DHX9 knockdown. DHX9 suppression resulted in a ~1.5-fold increase in cell death in *p53^−/−^*Eμ- *Myc* lymphomas, compared to a ~3-fold increase in *TSC2^+/−^*Eμ-*Myc* lymphomas (Figure 2A); this difference in the extent of cell death induced is consistent with the slower kinetics of depletion exhibited by the *p53^−/−^*Eμ-*Myc* lymphomas in the competition assay (Figure 1A). In the MEFs, neither the INK4A^−/−^ nor the p53^−/−^ MEFs showed an increase in cell death (Figure 2B). The HCT116 p53^−/−^ cells exhibited a 2.9–3.5-fold increase in cell death, compared to a 4.4–4.9 fold increase in p53^+/+^ cells (Figure 2C), which again, was consistent with the slight difference in kinetics observed in the competition assay. Given that the MEFs showed no induction of cell death upon DHX9 suppression, we carried out cell cycle analysis on the three different cell types following transduction with control or DHX9 shRNAs. Indeed, the INK4A^−/−^ MEFs exhibited a marked increase (~24%) in the percentage of cells in G0/G1 phase, and a ~12% decrease in the number of cells in both the S and G2/M phases upon DHX9 suppression, indicating a G0/G1 growth arrest. In the case of the p53^−/−^ MEFs, changes in cell cycle distribution following DHX9 knockdown were smaller, with a ~15% increase in the number of cells in G0/G1 phase, a 10% decrease in the cells in S phase, and a ~5% decrease in the number of cells in the G2/M phase (Figure 2E). This correlated with the slower kinetics of depletion in the competition assay for the p53^−/−^ MEFs (Figure 1B). Neither the HCT116 p53^+/+^ nor the HCT116 p53^−/−^ cells displayed any significant changes in cell cycle distribution, suggesting that depletion of the shDHX9-expressing cells was likely solely due to induction of cell death rather than growth arrest (Figure 2F). Both *TSC2^+/−^*Eμ-*Myc* and *p53^−/−^* Eμ-*Myc* lymphomas underwent a slight S-phase arrest upon loss of DHX9, with a small (3.5–4.6%) increase in the percentage of S-phase cells and a concomitant decrease in G2/M-phase cells (Figure 2D); however, cell death appeared to be the primary mechanism by which DHX9 suppression reduced the proliferative fitness of the lymphomas. These results indicate that irrespective of the p53 status, DHX9 suppression may elicit a cell death or cell cycle arrest response, or a combination of both, depending on the cellular context.

### DHX9 suppression activates p53 targets in both p53-wildtype and p53-null systems

**Figure 3:**
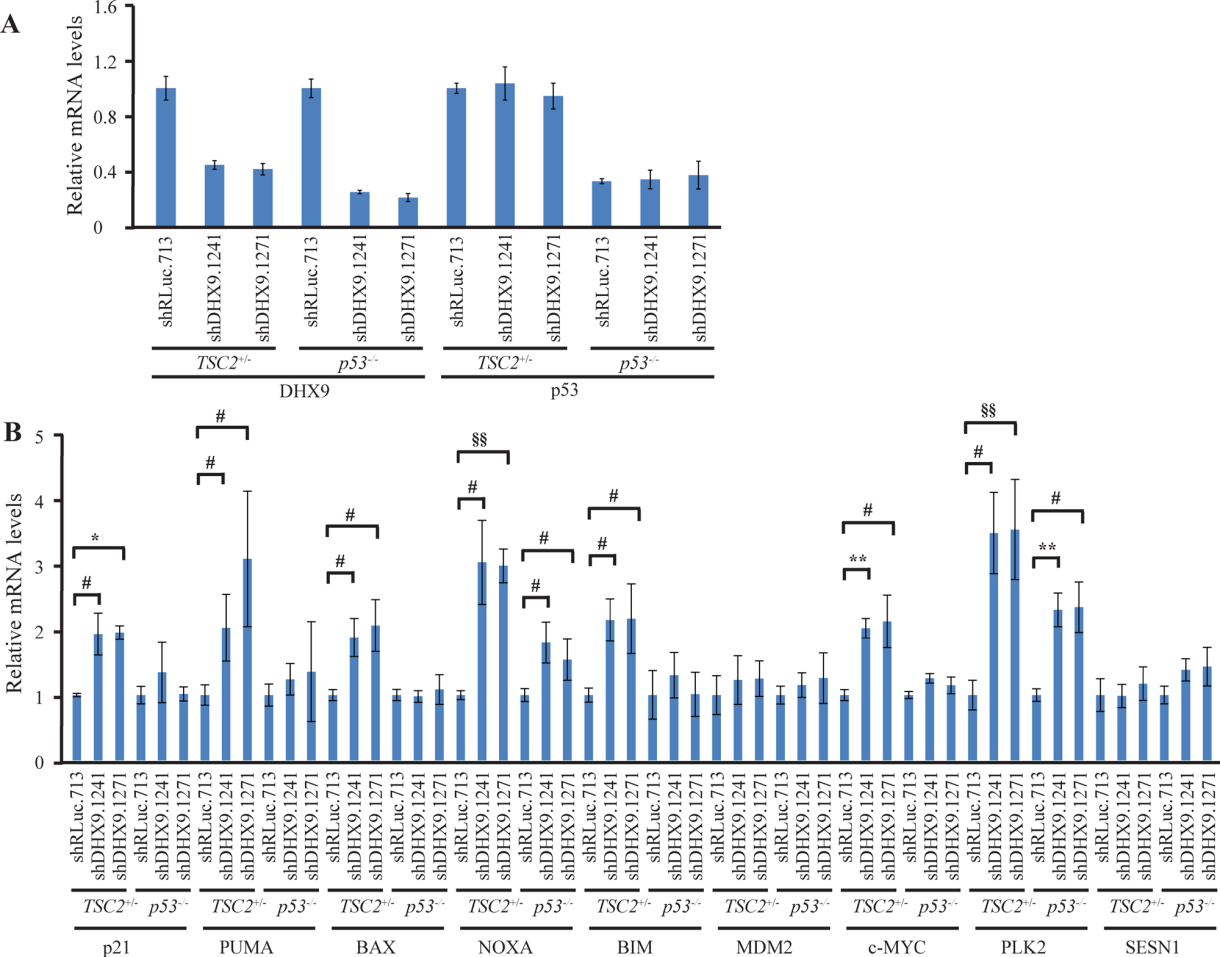
Consequences of DHX9 knockdown on p53 targets in TSC2^+/−^Eμ-Myc (p53^+/+^) and p53^−/−^ Eμ-Myc lymphoma cells (**A**) Quantitative RT-PCR analysis documenting DHX9 knockdown and p53 levels in *TSC2^+/−^*Eμ-*Myc* (p53^+/+^) and *p53^−/−^* Eμ-*Myc* lymphomas. The indicated cell lines were transduced with control shRLuc.713 or DHX9 shRNAs and harvested 6 days post-infection. mRNA levels were normalized to GAPDH and the mRNA levels of the shDHX9 samples were then normalized to that of the shRLuc.713 sample for each cell line. *N* = 3 ± SEM. (**B**) Quantitative RT-PCR analysis of p53 transcriptional targets in *TSC2^+/−^*Eμ-*Myc* (p53^+/+^) and *p53^−/−^* Eμ-*Myc* lymphomas. The analysis was performed 6 days post-transduction with control shRLuc.713 or DHX9 shRNAs. mRNA levels for each cell line and target were normalized as in (A). N = 3 ± SEM. Significant differences between shDHX9 and shRLuc.713 samples (where *p* ≤ 0.05) are indicated as follows: ^#^*p* ≤ 0.05, ^§^*p* ≤ 0.01, *p ≤ 0.005, ^##^*p* ≤ 0.001, ^§§^*p* ≤ 0.0005, ^*^p ≤ 0.0001.

**Figure 4:**
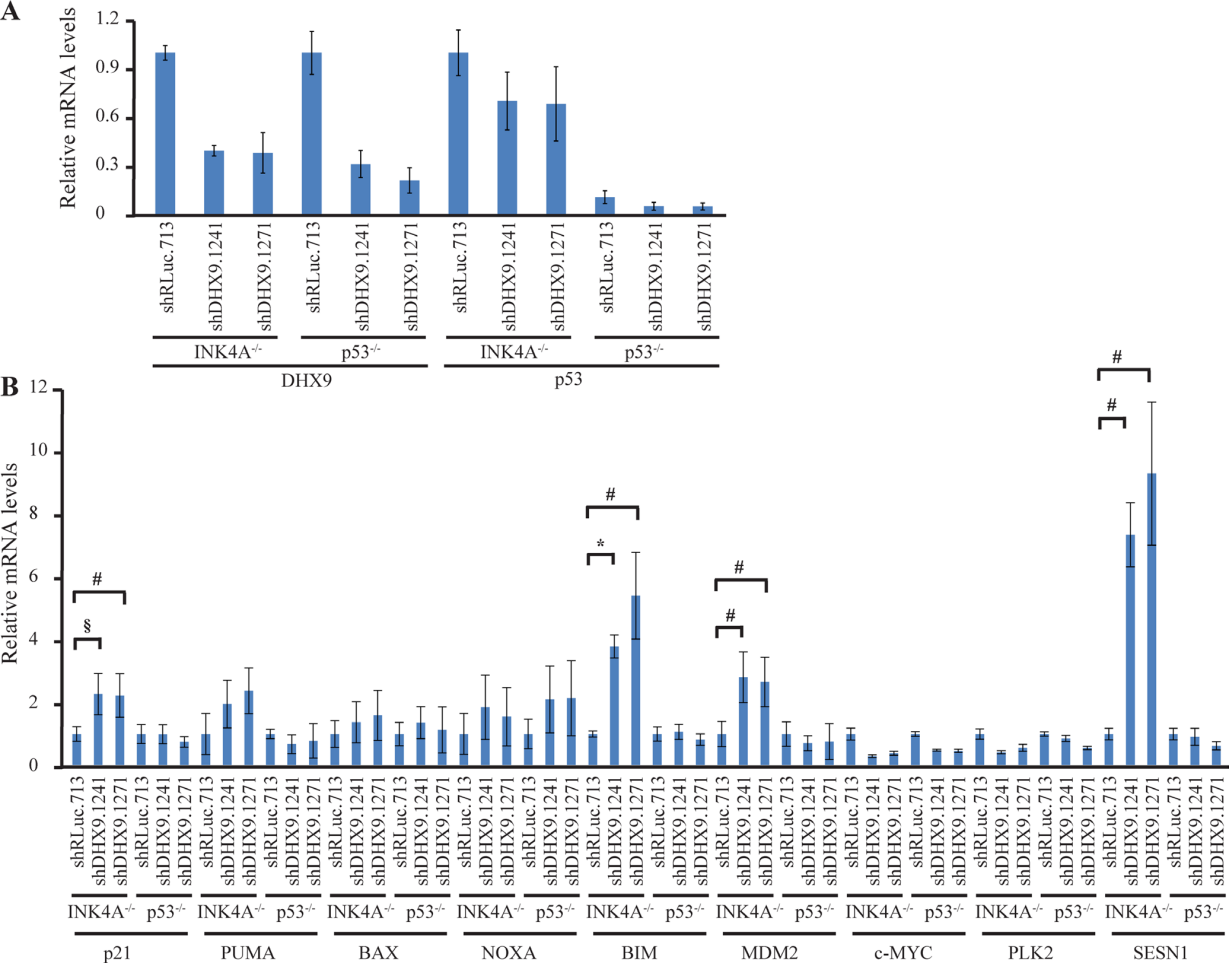
Consequences of DHX9 knockdown on p53 targets in INK4A^−/−^ (p53^+/+^) and p53^−/−^ MEFs (**A**) Quantitative RT-PCR analysis showing DHX9 knockdown and p53 levels in INK4A^−/−^ (p53^+/+^) and p53^−/−^ MEFs. The indicated cell lines were transduced with control shRLuc.713 or DHX9 shRNAs and harvested 6 days post-infection. mRNA levels were normalized to GAPDH and the mRNA levels of the shDHX9 samples were then normalized to that of the shRLuc.713 sample for each cell line. *N* = 3 ± SEM. (**B**) Quantitative RT-PCR analysis of p53 transcriptional targets in INK4A^−/−^ (p53^+/+^) and p53^−/−^ MEFs. The analysis was performed 6 days post-transduction with control shRLuc.713 or DHX9 shRNAs. mRNA levels for each cell line and target were normalized as in (A). *N* = 3 ± SEM. Significant differences between shDHX9 and shRLuc.713 samples (where *p* ≤ 0.05) are indicated using the same key as in Figure 3.

**Figure 5:**
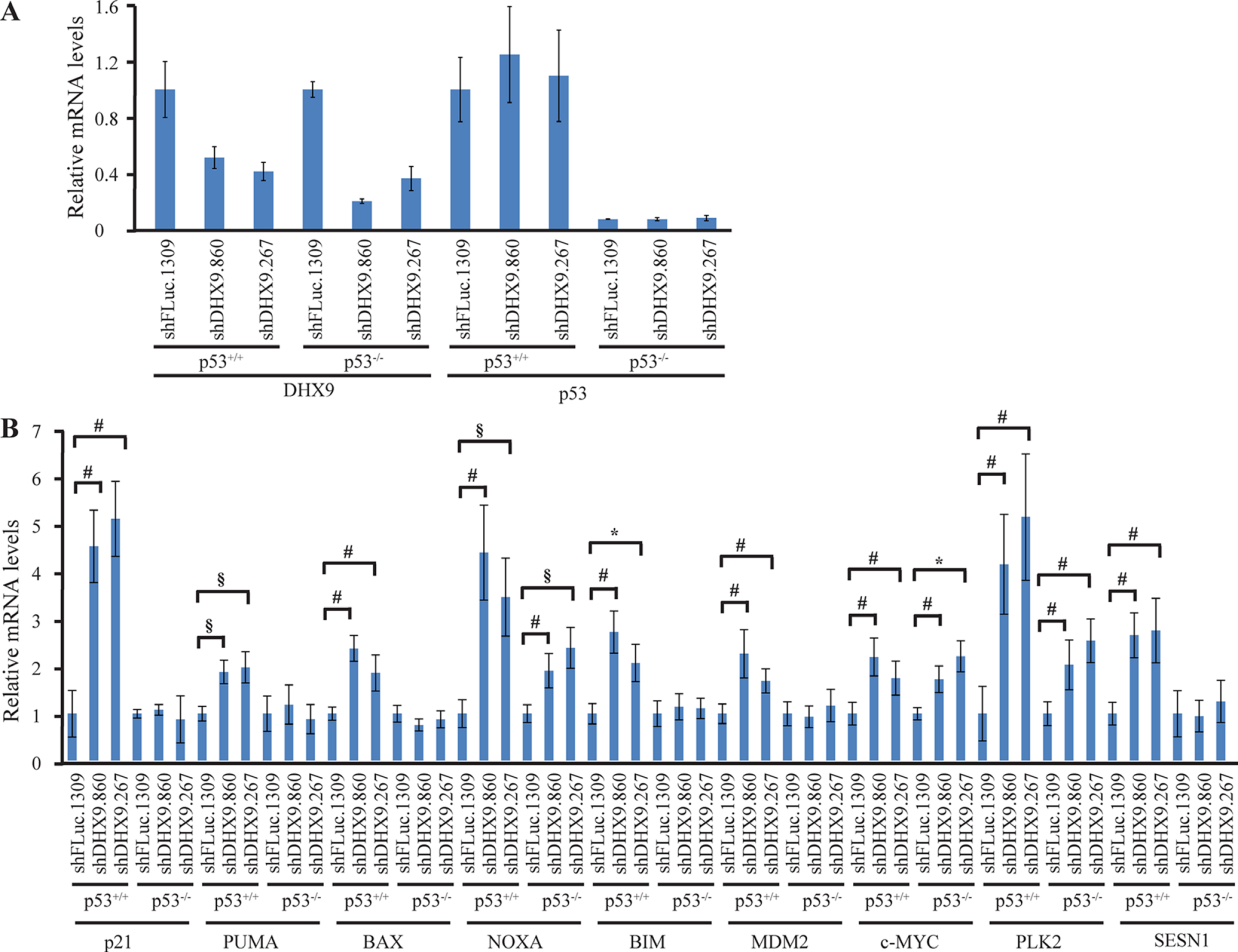
Consequences of DHX9 knockdown on p53 targets in HCT116 p53^+/+^ and p53^−/−^ cells (**A**) Quantitative RT-PCR analysis showing DHX9 knockdown and p53 levels in HCT116 p53^+/+^ and p53^−/−^ cells. The indicated cell lines were transduced with control shFLuc.1309 or DHX9 shRNAs and harvested 6 days post-infection. mRNA levels were normalized to GAPDH and the mRNA levels of the shDHX9 samples were then normalized to that of the shFLuc.1309 sample for each cell line. *N* = 3 ± SEM. (**B**) Quantitative RT-PCR analysis of p53 transcriptional targets in HCT116 p53^+/+^ and p53^−/−^ cells. The analysis was performed 6 days post-transduction with control shFLuc.1309 or DHX9 shRNAs. mRNA levels for each cell line and target were normalized as in (A). *N* = 3 ± SEM. Significant differences between shDHX9 and shFLuc.1309 samples (where *p* ≤ 0.05) are indicated using the same key as in Figure 3.

**Figure 6:**
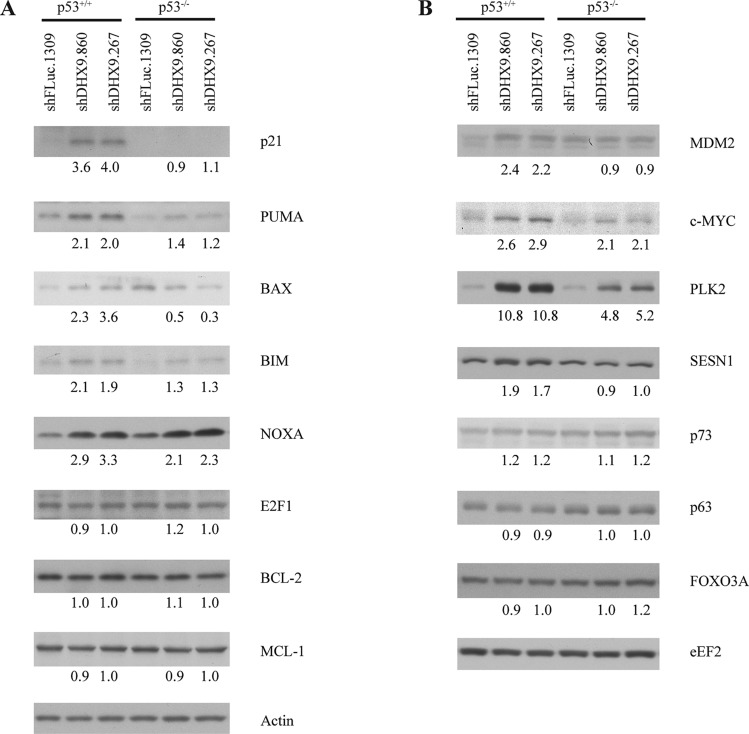
Consequences of DHX9 knockdown on protein levels of p53 targets in HCT116 p53^+/+^ and p53^−/−^ cells Western blot analysis of p53 transcriptional targets in HCT116 p53^+/+^ and p53^−/−^ cells. Cells were harvested 6 days post-transduction and extracts were fractionated on (**A**) 15% and (**B**) 8% SDS-PAGE gels. In each panel ((A) and (B)), all probings were performed on the same blot. Actin and eEF2 are used as loading controls. Quantitation of intensity levels of the proteins in the shDHX9 samples relative to the shFLuc.1309 samples are indicated beneath each band.

To better understand how loss of DHX9 elicits a cell death or growth arrest response in p53-wildtype and p53-null systems, we quantified the relative mRNA levels of a set of known, previously validated p53 transcriptional target genes [16, 23] using quantitative RT-PCR analysis. Nine p53 target genes were analyzed in *TSC2^+/−^*Eμ-*Myc* and *p53^−/−^* Eμ-*Myc* lymphomas, INK4A^−/−^ and p53^−/−^ MEFs, and HCT116 p53^+/+^ and p53^−/−^ cells, six days after transduction with control or DHX9 shRNAs. Amongst these were the cyclin dependent kinase inhibitor p21, pro-apoptotic BCL-2 family proteins PUMA, BAX, NOXA, BIM, the c-MYC oncogene, and other targets MDM2, PLK2, and SESN1. DHX9 knockdown and p53 levels were validated in the RNA samples for each cell type (Figures 3A, 4A, and 5A). p53 mRNA levels were not elevated in response to DHX9 knockdown, suggesting that the observed increase in p53 protein (Figure 1D–1F) is likely due to a post-transcriptional response. DHX9 suppression in *TSC2^+/−^*Eμ-*Myc* cells resulted in elevated levels of p21, PUMA, BAX, NOXA, BIM, c-MYC, and PLK2. Of these, NOXA and PLK2 were also elevated in *p53^−/−^* Eμ-*Myc* lymphomas; however, the magnitude of induction for both genes was approximately 1.5–2-fold less than that experienced in the *TSC2^+/−^*Eμ-*Myc* lymphomas (Figure 3B). p21, BIM, MDM2, and SESN1 levels were significantly increased upon DHX9 knockdown in INK4A^−/−^ MEFs, but none of the p53 targets tested appeared to be activated in p53^−/−^ MEFs (Figure 4B). p21, PUMA, BAX, NOXA, BIM, MDM2, c-MYC, PLK2 and SESN1 transcript levels increased following DHX9 suppression in HCT116 p53^+/+^ cells, and the HCT116 p53^−/−^ cells exhibited significant increases in NOXA, c-MYC, and PLK2 levels (Figure 5B). Similar to what was observed in the lymphomas, the magnitude of the increases in NOXA and PLK2 was approximately 1.5–2-fold less in the HCT116 p53^−/−^ cells compared to HCT116 p53^+/+^ cells. We also examined the protein levels of the p53 targets in the HCT116 cells. Immunoblot analysis showed increases in the protein levels of the p53 targets which corresponded to the mRNA data in HCT116 cells (Figure 6). In particular, NOXA, c-MYC, and PLK2 protein levels were significantly upregulated in both the p53^+/+^ and p53^−/−^ HCT116 cells. These results illustrate that in cells harboring functional p53, DHX9 suppression activates a transcriptional program consisting of several targets known to lead to cell death or cell cycle arrest. In the absence of p53, a subset of classic p53 targets is also activated and this may contribute to the cell death response elicited in these cells.

Having observed an effect on the expression of pro-apoptotic genes following DHX9 suppression, it is also possible that down-regulation of anti-apoptotic genes played a role in inducing cell death. Indeed, p53 can mediate transcriptional repression of certain anti-apoptotic proteins [24–28]. We examined the transcript levels of three known anti-apoptotic p53 targets: BCL-2, MCL-1, and survivin. We observed no change in expression of these genes upon DHX9 suppression in the three different cell lines, with or without functional p53 (Supplementary Figure 3A–3C). We also observed no change in the protein levels of BCL-2 and MCL-1 (Figure 6A). This suggests that induction of cell death in response to DHX9 knockdown in the lymphomas and HCT116 cells is likely a consequence of increased expression of pro-apoptotic factors rather than downregulation of anti-apoptotic proteins.

In the absence of functional p53, it has been reported that other transcriptional factors may activate p53 targets (see DISCUSSION). To gain further insight into the mechanism by which the p53 targets may be activated in the p53-null cells, we examined the protein levels of several known alternative transcription factors in HCT116 cells: the p53 family members p73 and p63, c-MYC, E2F1, and FOXO3A (Figure 6). Of these, only c-MYC levels showed an increase (~2-fold) in p53^−/−^ HCT116 cells. Hence, c-MYC may contribute to induction of cell death in the p53-null setting.

## DISCUSSION

The tumor suppressor p53 plays a central regulatory role in cell cycle progression, apoptosis, and DNA repair. It transcriptionally activates genes with a wide range of functions in response to cellular stresses such as DNA damage, reactive oxygen species, and replication stress, allowing the cell to arrest or undergo apoptosis to prevent aberrant replication and genomic instability [29]. p53 transcriptional targets include the cyclin-dependent kinase inhibitor p21 [30, 31], the tumor suppressor PTEN [32], members of the BCL-2 family of pro-apoptotic factors such as PUMA, NOXA, and BAX [33–36], as well as components of the apoptotic effector machinery (e.g. APAF-1 and caspase-6) [37–40]. It is among the most frequently mutated gene in human cancer, with over 50% of cancers harboring a defect in p53 [41, 42]. Since many traditional genotoxic agents act through p53 to induce apoptosis or cell cycle arrest, this poses a problem for chemotherapy because it restricts the use of these agents to settings where p53 is functional. Investigating means of eliciting a cell cycle arrest or cell death response in the absence of functional p53 is therefore of great therapeutic interest.

Previous studies from our research group have supported the notion of inhibiting DHX9 as a chemotherapeutic approach, primarily in p53-wildtype settings. In the present study, we assessed the consequences of suppressing DHX9 in p53-deficient cells and compared the outcome to that achieved in p53-wildtype scenarios. We demonstrated that p53-null mouse lymphomas, MEFs, and HCT116 cells are susceptible to DHX9 suppression.

We chose to examine whether common p53 targets were also being activated in p53-null cells. In *TSC2^+/−^*Eμ-*Myc* lymphomas, DHX9 suppression resulted in elevated levels of p21, PUMA, BAX, NOXA, BIM, c-MYC, and PLK2. This is consistent with activation of an apoptotic program previously observed in mouse lymphomas upon DHX9 suppression [16]. We found that NOXA and PLK2 were also elevated in *p53^−/−^* Eμ-*Myc* lymphomas. A similar situation was observed with the p53^+/+^ and p53^−/−^ HCT116 cells: induction of p21, PUMA, BAX, NOXA, BIM, MDM2, c-MYC, PLK2, and SESN1 mRNA in the p53^+/+^ cells and of NOXA, c-MYC, and PLK2 in the p53^−/−^ cells. Upregulation of these genes was confirmed at the protein level in the HCT116 cells (Figure 6). The fact that we observed increased levels of NOXA and PLK2 in both p53-null lymphomas and HCT116 cells suggests that these two proteins may be involved in common p53-independent pathways of activating programmed cell death. The smaller magnitude of increase in NOXA and PLK2 levels in the p53-null cells, as well as the observation that fewer p53 targets were activated in the p53-null cells compared to the p53-wildtype cells, may be responsible for the slower kinetics of depletion and lower levels of cell death induced upon DHX9 loss in the p53-null cells. DHX9 suppression in INK4A^−/−^ MEFs resulted in increased levels of p21, BIM, MDM2, and SESN1, all of which are known mediators of cell cycle arrest [30, 31, 43–46]. In contrast to what was observed in the p53-wildtype lymphomas and HCT116 cells, no changes in pro-apoptotic factors PUMA, BAX, and NOXA were exhibited by the INK4A^−/−^ MEFs. This is consistent with the different cell fates (cell cycle arrest in the MEFs versus apoptosis in the lymphomas and HCT116 cells) resulting from DHX9 suppression (Figure 2).

While activation of p53 signaling is the canonical pathway by which apoptosis or cell cycle arrest is triggered, these processes have also been documented to occur in a p53-independent manner. Studies have shown that many *bona fide* p53 targets can be activated in p53-deficient settings. In some cases, p53-independent activation occurs through upregulation by other transcription factors. For example, aside from p53, p21 transcription can also be activated by E2F1, E2F3, SP1, SP3, members of the Krüppel-like transcription factor (KLF) family (e.g. KLF4 and KLF6), CDX2, BETA2, MYOD1, and a variety of nuclear receptors [47–51]. Much attention has been given to the p53 family member, p73, and its role in cell cycle control and apoptosis. p73 shares significant structural homology with p53, binds to canonical p53 promoter elements, and can transactivate many p53-dependent promoters [52–54]. Although p73 is known to function cooperatively with p53 and another p53 homolog, p63 [55], it can also activate p53 targets independently of p53. Notably, p73 can transcriptionally activate NOXA, PUMA, and p21 in p53-deficient cells in response to a variety of genotoxic stimuli [56–58]. p21, NOXA, PUMA, and BIM are also transactivated by the transcription factors E2F1 [59–62] and FOXO3A [63–66] in a p53-independent manner. c-MYC is another activator of NOXA [67]. Hence, cell cycle arrest or programmed cell death via the intrinsic apoptotic pathway can take place in p53-deficient settings by virtue of activation of cell cycle or apoptotic proteins by these other transcription factors. Examination of the protein levels of the aforementioned possible alternative transcription factors showed that c-MYC levels were increased in p53^−/−^ HCT116 cells (Figure 6). Given that activation of NOXA by c-MYC has been previously reported [67], it is possible that c-MYC may contribute to p53-independent induction of cell death by transcriptionally activating NOXA. While none of the other known alternative transcription factors showed activation upon DHX9 suppression in the p53-null setting, it is possible that yet-unidentified transcription factors may also contribute to the apoptotic response. In addition, cell cycle arrest may proceed via activation of the p16-RB1 pathway independently of both p53 and p21 [68], which may be a possibility for the p53^−/−^ MEFs given that none of the p53 targets examined showed any upregulation upon DHX9 suppression (Figure 4B). Taken together, our results suggest that DHX9-mediated cell death in the p53^−/−^ lymphomas and HCT116 cells may involve p53-independent upregulation of NOXA and PLK2, which may be activated by transcription factors other than p53.

We have previously shown that DHX9 suppression resulted in senescence in primary human diploid fibroblasts and synergized with ABT-737 to induce apoptosis in *Arf*^−/−^Eμ-*Myc*/Bcl-2 mouse lymphoma cells, in a p53-dependent manner [16, 17]. Here, we show that DHX9 loss can also have deleterious effects in p53-deficient cells. Taken together, these results indicate that the consequences of DHX9 suppression will be context-dependent. We have observed that DHX9 knockdown results in a cell death response in the majority of tumor cell lines but a growth arrest response in non-transformed cells. Loss of DHX9 also has a differential effect in mouse tissues *in vivo* versus cell lines *ex vivo*, as previously revealed [18]. It is therefore conceivable that p53 may be required for DHX9-mediated cell cycle arrest and senescence in non-transformed primary cells but not for a cell cycle arrest or apoptotic response in immortalized cell lines or tumor cells, which harbor significant differences in their biological wiring. Indeed, there are previously documented instances where a particular agent may cause p53-dependent apoptosis in one cellular context but p53-independent apoptosis in another. In one example, sepsis-induced apoptosis was found to be p53-dependent in thymocytes but p53-independent in splenocytes [69]. In another case, paclitaxel-mediated apoptosis was p53-dependent in EIA-transformed MEFs, but when the cells were simultaneously exposed to the cytokine tumor necrosis factor α (TNF-α), the effect became p53-independent [70]. In conclusion, our study supports the presence of a p53-independent mechanism of cell death and cell cycle arrest resulting from DHX9 inhibition. While further work is required to characterize this effect in greater depth, our results support the feasibility of targeting DHX9 as a chemotherapeutic approach in p53-deficient tumors.

## MATERIALS AND METHODS

### Cell lines and cell culture

HEK293T/17 cells (ATCC, Manassas, VA, USA), INK4A^−/−^ p53^+/+^ MEFs, and TSC2^+/+^ p53^−/−^ MEFs (a kind gift from Dr. David Kwiatkowski (Brigham and Women's Hospital, USA)) were maintained in DMEM (Multicell, St-Bruno, QC, Canada). HCT116 p53^+/+^ and HCT116 p53^−/−^ cells were maintained in McCoy 5A (Multicell). Media was supplemented with 10% fetal bovine serum (Multicell). *TSC2^+/−^*Eμ*−Myc* lymphoma cells were derived from tumors in *TSC2^+/−^* mice crossed with Eμ*-Myc* mice. *TSC2^+/−^*Eμ*-Myc* lymphomas retained wildtype p53, as determined by sequencing across all p53 coding exons and Western blot analysis following γ-irradiation [20]. Similarly, *p53^−/−^* Eμ*-Myc* and *Arf^−/−^* Eμ*-Myc* lymphomas were derived from tumors in *p53^−/−^* or *Arf^−/−^* mice crossed with Eμ*-Myc* mice. Lymphomas were cultured in B-cell media (45% DMEM, 45% Iscove's media, 55 mM β-mercaptoethanol, 10% fetal bovine serum) on irradiated INK4A^−/−^ MEF feeder layers.

### Plasmids, virus generation and transductions

For suppression of DHX9 in murine cell lines (MEFs and Eμ-*Myc* lymphomas), two independent shRNAs targeting mouse DHX9 (shDHX9.1241 and shDHX9.1271) and a control shRNA targeting renilla luciferase (shRLuc.713) were transduced into cells using the MSCV/LTR/miR30/PuroR-IRES-GFP (MLP) or MSCV/LTR/miR30/SV40-GFP (MLS-GFP) retroviral vectors. Retroviral infections were generated using ecotropic Phoenix packaging cells following established protocols (https://web.stanford.edu/group/nolan/_OldWebsite/retroviral_systems/retsys.html). For infections using MLP, stable integrants were selected using 2 μg/ml puromycin for at least 2 days after the final infection. For suppression of DHX9 in HCT116 cells, two shRNAs targeting human DHX9 (shDHX9.860 and shDHX9.267) and a control shRNA targeting firefly luciferase (shFLuc.1309) were transduced into cells using pPrime-PGK-Puro (Addgene, Cambridge, MA, USA). Lentiviral transduction was performed following published procedures [71]. All shRNAs in this study were cloned into the miR30 backbone of their corresponding vectors via unique XhoI and EcoRI restriction sites [72]. The guide strand sequences of the shRNAs have been previously published [18].

For generation of TSC2-knockdown murine cell lines, *p53^−/−^* Eμ-*Myc* or *Arf^−/−^* Eμ-*Myc* lymphomas were transduced with either an shRNA targeting TSC2 (shTSC2) or the shFLUC.1309 control, using the MSCV/LTR/miR30/SV40-mCherry (MLS-mCherry) retroviral vector. Cells were sorted for the mCherry+ expressing population. The guide strand sequence of the TSC2 shRNA is ^5′^GGCCCGATATGTGTTCTCCAAT^3′^.

### *Ex vivo* competition assays

*Ex vivo* competition assays were performed by transducing cells with MLS-GFP-based (for lymphomas and MEFs) or pPrime-PGK-puro-based (for HCT116 cells) shRNAs. The percentage of GFP-positive cells was measured 48h after the final infection (*t* = 0) using a GUAVA EasyCyte HT flow cytometer (Millipore, Billerica, MA, USA), and assessed every 2–3 days thereafter. Cell death was assessed by staining cells with 4 μg/ml propidium iodide (PI) and measuring the percentage of PI-positive cells.

### Cell cycle analysis

Cell cycle analysis was performed using ethanol fixation, acid denaturation, and propidium iodide (PI) staining as previously described [18]. Briefly, cells were harvested from a 6 cm plate, washed twice with PBS containing 1% BSA and 5 mM EDTA, resuspended in 50 μl PBS on ice, fixed with 1.25 ml 70% ethanol, and stored at –20^°^C until further processing. The fixed cells were then treated with 0.5% Triton X-100/ 2 M HCl, neutralized with 0.1 M sodium borate [pH 8.5], washed with PBS containing 1% BSA and 0.5% Triton X-100, and resuspended in 500 μL of PBS containing 5 μg/mL PI (Sigma). The cell cycle profile of the cells was assessed using a GUAVA EasyCyte HT flow cytometer (Millipore, Billerica, MA, USA).

### Immunoblot analysis

Protein extracts were prepared by lysing cells in RIPA lysis buffer (20 mM Tris-HCl [pH 7.5], 150 mM NaCl, 0.1% SDS, 1% NP40, 0.5% sodium deoxycholate, 1 mM β-glycerophosphate, 1 mM PMSF, 1 μg/ml leupeptin, 10 μg/ml aprotinin, and 2.5 μM pepstatin A). PVDF membranes were probed with the indicated primary antibodies and HRP-conjugated secondary antibodies (rabbit (711-035-152) or mouse (115-035-146) (Jackson ImmunoResearch, West Grove, PA, USA) and visualized using enhanced chemiluminescence (ECL) (Perkin Elmer, Waltham, MA, USA). Western blot quantification was performed using Image Studio Lite (LI-COR Biotechnology, Lincoln, NE, USA). The primary antibodies used in this study were: DHX9 (M99; SC Biotech (Dallas, TX, USA) for human and ab26271; Abcam (Cambridge, MA, USA) for mouse), p53 (DO-1; SC Biotech for human and NL-p53-505; Novocastra (Concord, ON, Canada) for mouse), α-actin (clone AC-15; Sigma, Oakville, ON, Canada), p21 (556430; BD Pharmingen, Franklin Lakes, NJ, USA), PUMA (#3041, ProSci, Poway, CA, USA), BIM (#202000, Millipore, Billerica, MA, USA), SESN1 (ab134091; Abcam, Cambridge, MA, USA), E2F1 (8G9; Novus Biologicals, Littleton, CO, USA), p73 (5B429; Novus Biologicals, Littleton, CO, USA), p63 (orb214808; Biorbyt, San Francisco, CA, USA). Antibodies for NOXA (D8L7U)(#14766), PLK2 (D5R2B)(#14812), FOXO3A (#9467), and eEF2 (#2332) were purchased from Cell Signaling (Danvers, MA, USA). Antibodies for c-MYC (N-262), MDM2 (SMP14), BAX (B-9), BCL-2 (C-2), MCL-1(S-19), and TSC2 (C-20) were purchased from SC Biotech (Dallas, TX, USA).

### Quantitative RT-PCR analysis

Total RNA was extracted from cells using TRIzol as per the manufacturer's instructions (Invitrogen, Carlsbad, CA, United States) six days after infection with shRNAs targeting DHX9 or the luciferase control. The RNA was treated with DNase I (Thermo Fisher Scientific, Waltham, MA, USA) and cDNA was generated using Superscript III Reverse Transcriptase (Invitrogen) as per the manufacturer's instructions. Quantitative RT-PCR was performed using the SsoFast EvaGreen Supermix reagent (Bio-Rad, Hercules, CA, USA) on a CFX96 Touch Real-Time PCR Detection System (Bio-Rad). The following primers were used for PCR amplification of mouse targets: DHX9 FWD-^5′^CCGAGGAGCCAACCTTAAAGA^3′^, REV-^5′^TGTCCAATTTCCATGAAGCCC^3′^; p53 FWD-^5′^G CGTAAACGCTTCGAGATGTT^3′^, REV-^5′^TTTTTATGG CGGGAAGTAGACTG^3′^; p21 FWD-^5′^CCTGGTGAT GTCCGACCTG^3′^, REV-^5′^CCATGAGCGCATCGCAATC ^3′^; PUMA FWD-^5′^ATGCCTGCCTCACCTTCATCT^3′^, REV-^5′^AGCACAGGATTCACAGTCTGGA^3′^; BAX FWD-^5′^TGAAGACAGGGGCCTTTTTG^3′^, REV-^5′^AAT TCGCCGGAGACACTCG^3′^; NOXA FWD-^5′^ACTGTG GTTCTGGCGCAGAT^3′^, REV-^5′^TTGAGCACACTCGT CCTTCAA^3′^; BIM FWD-^5′^GAGTTGTGACAAGTCAA CACAAACC^3′^, REV-^5′^GAAGATAAAGCGTAACAGT TGTAAGATAACC^3′^; MDM2 FWD-^5′^TGTCTGTGTC TACCGAGGGTG^3′^, REV-^5′^TCCAACGGACTTTAACA ACTTCA^3′^; c-MYC FWD-^5′^CAAATCCTGTACCTCG TCCGATTC^3′^, REV-^5′^CTTCTTGCTCTTCTTCAGAGT CGC^3′^; PLK2 FWD-^5′^GACTACTGCACCATAAGCA TG^3′^, REV-^5′^CTTCTGGCTCTGTCAACACCT^3′^; SESN1 FWD-^5′^GGCCAGGACGAGGAACTTG^3′^, REV-^5′^AAG GAGTCTGCAAATAACGCAG^3′^; BCL-2 FWD-^5′^GCTG GGATGCCTTTGTGGAACTA^3′^, REV-^5′^GGTATGCA CCCAGAGTGATGC^3′^; MCL-1 FWD-^5′^AAAGGCG GCTGCATAAGTC^3′^, REV-^5′^TGGCGGTATAGGTCGTC CTC^3′^; survivin FWD-^5′^GAGGCTGGCTTCATCCACT G^3′^, REV-^5′^CTTTTTGCTTGTTGTTGGTCTCC^3′^; and GAPDH FWD-^5′^AGGTCGGTGTGAACGGATTTG^3′^, REV-^5′^GGGGTCGTTGATGGCAACA^3′^.

The following primers were used for PCR amplification of human targets: DHX9 FWD ^5′^CAG GAGAGAGAGTTACTGCCT^3′^, REV-^5′^CTCTGCTGC TCGGTCATTCTG^3′^; p53 FWD ^5′^CAGCACATGACGG AGGTTGT^3′^, REV-^5′^TCATCCAAATACTCCACACGC ^3′^; p21 FWD ^5′^CGATGGAACTTCGACTTTGTCA^3′^, REV-^5′^GCACAAGGGTACAAGACAGTG^3′^; PUMA FWD ^5′^CAGACTGTGAATCCTGTGCT^3′^, Rev-^5′^ACA GTATCTTACAGGCTGGG^3′^; BAX FWD ^5′^AAGAAGC TGAGCGAGTGT^3′^, REV-^5′^GGAGGAAGTCCAATGTC ^3′^; NOXA FWD ^5′^GCTGGAAGTCGAGTGTGCTA^3′^, REV-^5′^CCTGAGCAGAAGAGTTTGGA^3′^; BIM FWD ^5′^TGGCAAAGCAACCTTCTGATG^3′^, REV-^5′^GCAGG CTGCAATTGTCTACCT^3′^; MDM2 FWD ^5′^GCAGTGA ATCTACAGGGACGC^3′^, REV-^5′^ATCCTGATCCAACC AATCACC^3′^; c-MYC FWD ^5′^AATGAAAAGGCCCCC AAGGTAGTTATCC^3′^, REV-^5′^GTCGTTTCCGCAACAA GTCCTCTTC^3′^; PLK2 FWD-^5′^TCAGCAACCCAGCA AACACAGG^3′^, REV-^5′^TTTCCAGACATCCCCGAAG AACC^3′^; SESN1 FWD- ^5′^CTACATTGGAATAATGGCTG CGG^3′^, REV- ^5′^AGGTCTATGGGCTAACACTTTGT^3′^; BCL-2 FWD-^5′^ GGTGGGGTCATGTGTGTGG ^3′^, REV-^5′^CGGTTCAGGTACTCAGTCATCC^3′^; MCL-1 FWD ^5′^AAGCCAATGGGCAGGTCT^3′^, REV-^5′^TGTCCAGT TTCCGAAGCAT^3′^; survivin FWD-^5′^AGAACTGGCCC TTCTTGGAGG ^3′^, REV-^5′^ CTTTTTATGTTCCTCTAT GGGGTC ^3′^; and GAPDH FWD-^5′^GAAGGTGAAGGT CGGAGTC^3′^, REV-^5′^GAAGATGGTGATGGGATTC^3′^.

### Generation of CRISPR-edited INK4A^−/−^ MEFs

INK4A^−/−^MEFs were transduced with sgRNAs targeting p53 (sgp53-1) or the ROSA control (sgROSA) as previously described [73], using the pQCX/sgRNA/Cas9/mCherry (QCiC) retroviral vector. Cells were sorted for a pure mCherry+ population and sgp53-transduced cells were treated with 10 μM Nutlin-3a to select for cells harboring editing at the p53 locus. To verify editing, T7 endonuclease cleavage assays and colony formation assays were performed as previously described [73].

### Statistical analysis

Statistical analysis was carried out using GraphPad Prism (v. 5.03, GraphPad Software Inc., La Jolla, CA, USA) and data is shown as mean ± SEM. Statistically significant differences were determined using the unpaired two-tailed *t*-test and represented as *p*-values.

## SUPPLEMENTARY MATERIALS FIGURES


