# Supplementary material for: Dependence of p53-deficient cells on the DHX9 DExH-box helicase
Source: Oncotarget. 2017 Mar 3;8(19):30908–21. doi: 10.18632/oncotarget.15889 (PMC5458177; doi:10.18632/oncotarget.15889)
Supplement: Supplementary file 1 [file oncotarget-08-30908-s001.pdf]

Dependence of p53-deficient cells on the DHX9 DExH-box helicase

Supplementary Materials

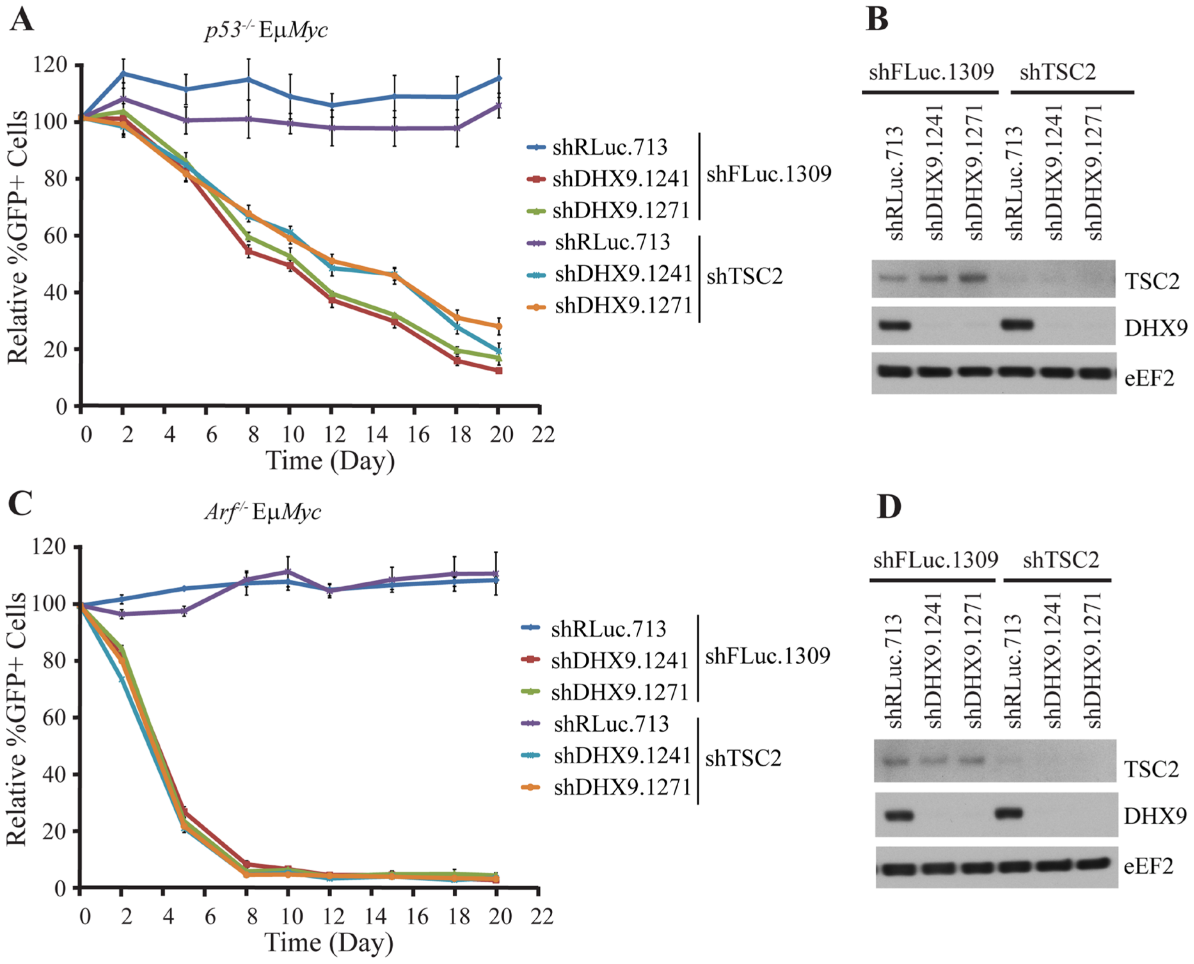

Supplementary Figure 1: TSC2 status does not significantly affect cell death in response to DHX9 suppression. *Ex vivo* competition assay with (**A**) *p53*$^{-/-}$Eμ-*Myc* and (**C**) *Arf*$^{-/-}$Eμ-*Myc* lymphomas. Cells were first infected with shRNAs in the MLS-mCherry vector targeting TSC2 or the firefly luciferase (FLuc) neutral control, sorted for a pure mCherry+ population, then subsequently infected with shRNAs targeting DHX9 or the renilla luciferase (RLuc) neutral control. The relative %GFP was monitored over time. The experiment was started 48 hours after the final infection (t = Day 0). $N = 3 \pm$ SEM. (**B**, **D**) Western blot analysis of extracts from the cell lines shown in (A) and (C) respectively.

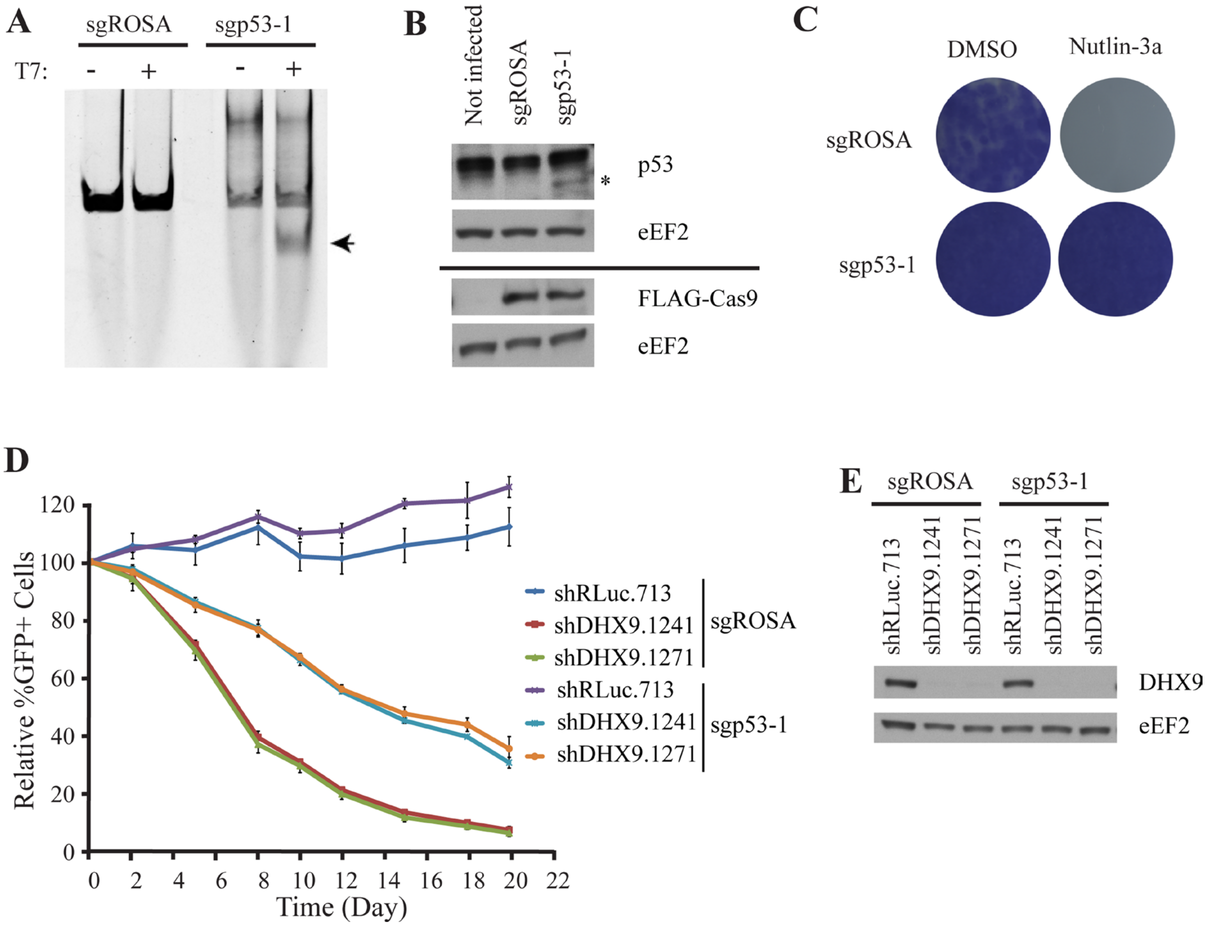

Supplementary Figure 2: DHX9 suppression reduces cellular fitness in isogenic p53-wildtype and p53-deficient INK4A$^{-/-}$ MEFs. (**A**) T7 endonuclease assay of DNA isolated from INK4A$^{-/-}$ MEFs transduced with sgRNAs targeting ROSA or p53 and exposed to 10 µM Nutlin-3a for 10 days. The arrowhead denotes the expected T7 endonuclease cleavage product. (**B**) Western blot documenting Cas9 and p53 expression in sgROSA and sgp53-infected INK4A$^{-/-}$ MEFs. Solid bar indicates that a different set of Western blots were probed. The asterisk denotes the position of a p53 truncated product only present in sgp53-transduced cells. (**C**) Colony formation assay of INK4A$^{-/-}$ MEFs transduced with sgROSA and sgp53-1. Five-thousand cells were seeded in each well of a 6-well plate, treated with DMSO or 10 µM Nutlin-3a, and stained with crystal violet 12 days later. (**D**) *Ex vivo* competition assay in INK4A$^{-/-}$ MEFs transduced with sgROSA or sgp53-1. Cells were infected with shRNAs targeting DHX9 or a neutral control and the relative %GFP monitored over time. The experiment was started 48 hours after the final infection (t = Day 0). $N = 3 \pm$ SEM. (**E**) Western blot analysis of extracts from (D).

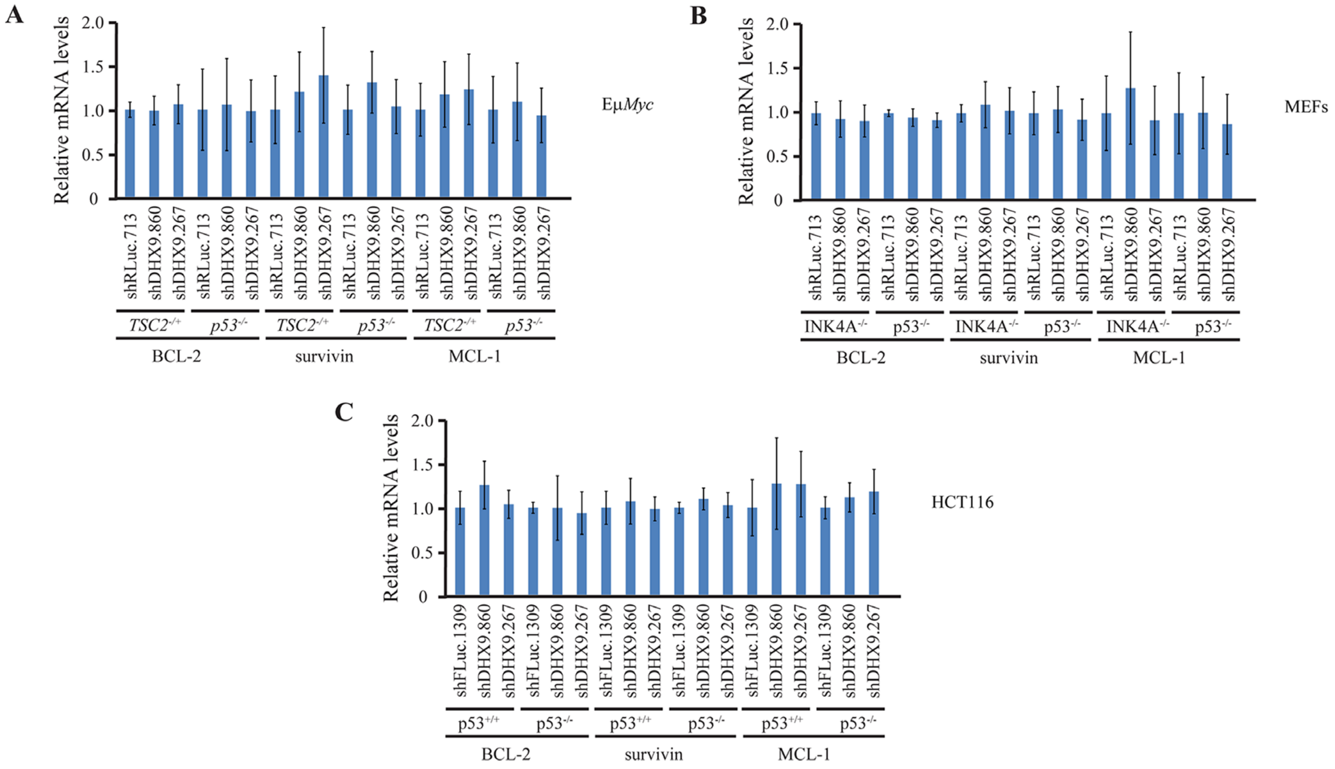

Supplementary Figure 3: Consequences of DHX9 knockdown on anti-apoptotic p53 targets in p53-wildtype and p53-null systems. Quantitative RT-PCR analysis of BCL-2, survivin, and MCL-1 was performed 6 days post-transduction with control or DHX9 shRNAs in (**A**) *TSC2*$^{+/-}$Eμ-*Myc* (p53$^{+/+}$) and *p53*$^{-/-}$ Eμ-*Myc* lymphomas, (**B**) INK4A$^{-/-}$ (p53$^{+/+}$) and p53$^{-/-}$ MEFs, and (**C**) HCT116 p53$^{+/+}$ and HCT116 p53$^{-/-}$ cells. mRNA levels were normalized to GAPDH and the mRNA levels of the shDHX9 samples were then normalized to that of the control (shRLuc.713 or shFLuc.1309) sample for each cell line. $N = 3 \pm$ SEM.
